# Case report: A highly active refractory myasthenia gravis with treatment of telitacicept combined with efgartigimod

**DOI:** 10.3389/fimmu.2024.1400459

**Published:** 2024-05-10

**Authors:** Chaoyue Zhang, Yangtao Lin, Qianjin Kuang, Hongjin Li, Qilong Jiang, Xiaojun Yang

**Affiliations:** The First Affiliated Hospital of Chinese Medicine, Guangzhou University of Chinese Medicine, Guangzhou, China

**Keywords:** myasthenia gravis, telitacicept, efgartigimod, highly active, refractory, case report

## Abstract

There is always a lack of effective treatment for highly active refractory generalized myasthenia gravis (GMG). Recently, telitacicept combined with efgartigimod significantly reduces circulating B cells, plasma cells, and immunoglobulin G, which brings promising therapeutic strategies. We report a case of a 37-year-old female patient with refractory GMG, whose condition got significant improvement and control with this latest treatment after multiple unsuccessful therapies of immunosuppressants. The new combination deserves further attention in the therapeutic application of myasthenia gravis.

## 1 Introduction

Myasthenia gravis (MG) is a rare autoimmune neuromuscular junction disorder characterized by autoantibody-mediated and complement-involved mechanisms (1, 2). In addition to the most common acetylcholine receptor antibodies (AChR-Ab), studies have progressively identified muscle-specific tyrosine kinase antibodies (MuSK-Ab), lipoprotein receptor–related protein 4 antibodies, ryanodine receptor antibodies (RyR-Ab), titin antibodies, and other pathogenic antibodies (2). Although conventional treatments based on acetylcholinesterase inhibitors, corticosteroids, and immunosuppressants are usually effective, patients continue to experience recurrent exacerbations and other safety concerns related to long-term immunosuppressants (3). Therefore, it is an urgent issue to search for safe and effective emerging therapies.

The 2023 Germany’s guideline (4) for MG has proposed a new concept about highly active GMG (including “refractory” MG), which can be defined that moderate/high Myasthenia Gravis Foundation of America (MGFA) status (≥MGFA IIb) and/or at least two recurrent severe exacerbations/myasthenic crises with the need for therapeutic intervention. Neonatal Fc receptor modulators (currently efgartigimod) are recommended for AChR-Ab–positive status. As the FcRn inhibitor, efgartigimod blocks immunoglobulin G (IgG) binding to FcRn, resulting in IgG degradation (5). In addition, as an inhibitor for the activity of BLyS (B lymphocyte stimulator) and APRIL (a proliferation-inducing ligand), telitacicept was originally designed to diseases such as systemic lupus erythematosus (6) and rheumatoid arthritis (7). However, according to its mechanism that achieves multistage inhibition of B-cell maturation and differentiation (8), it can be a potential efficacy treatment for patients with MG.

We herein report a young female patient with highly active refractory GMG after multiple unsuccessful therapies of immunosuppressants (adequate quantity and course), who was positive for AChR-Ab (early onset) and RyR-Ab and acquired recurrent intravenous immunoglobulin (IVIg) and plasma exchange (PLEX) after recurrent myasthenic crises. With telitacicept combined with efgartigimod, this patient’s condition got significant improvement and control.

## 2 Case description

**Figure 1:**
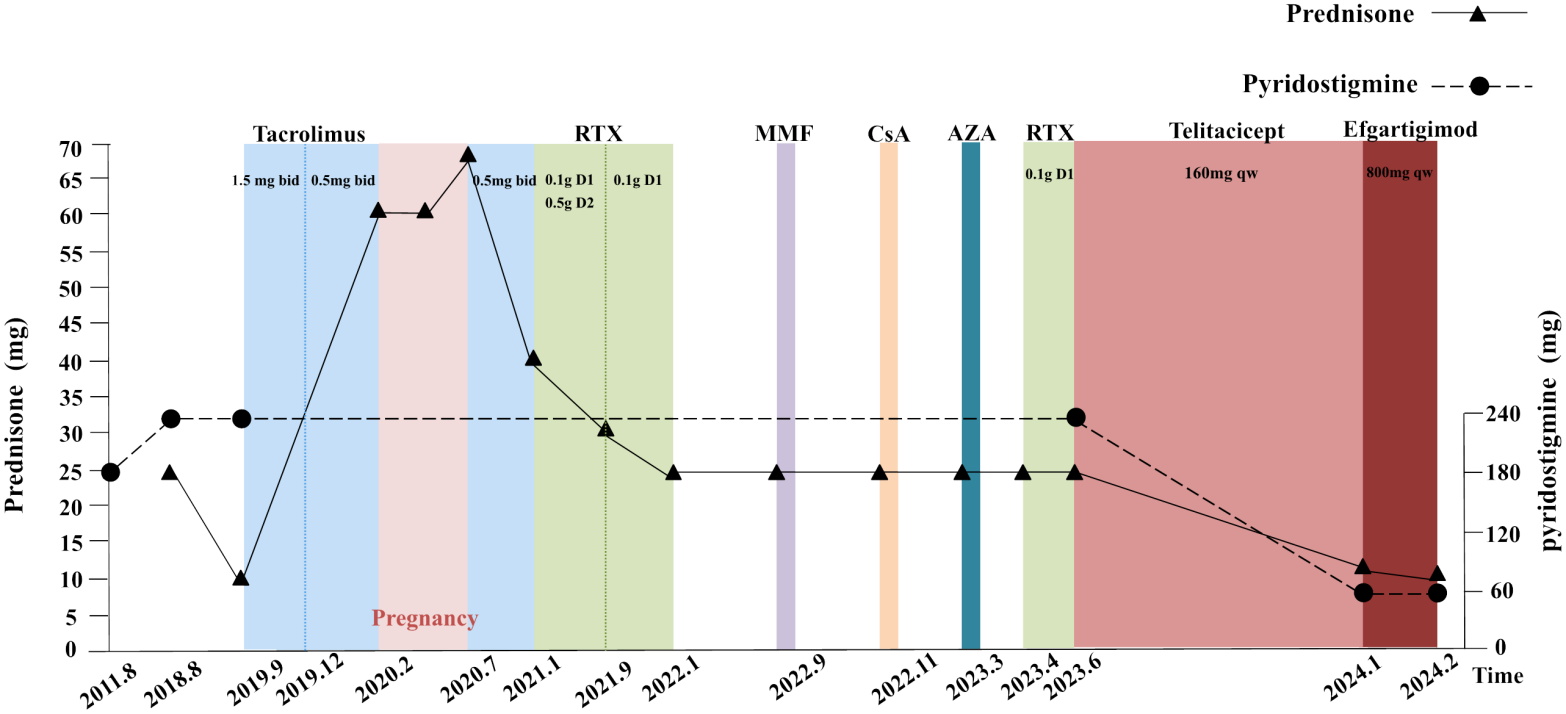
Previous therapeutic strategies and the dose of prednisone and pyridostigmine. Each time point represents a MC, which used rapid treatment of IVIg, PLEX, and high-dose steroid (before June 2023). Abbreviations: MC, myasthenic crises; RTX, rituximab; MMF, mycophenolate mofetil; CsA, cyclosporine A; AZA, azathioprine; IVIg, intravenous immunoglobulin; PLEX, plasma exchange. Details: The dose of pyridostigmine was 60 mg ter in die (tid) during 2011 to 2018 and increased to 60 mg quater in die (qid) until June 2023. The prednisone’s dosage was 25 mg qd in August 2018 and declined to 10 mg quaque die (qd) when combining tacrolimus (1.5 mg bid) in September 2019. In February 2020, the patient found an intrauterine pregnancy of 7+ weeks and chose to continue the pregnancy. She discontinued tacrolimus, and the dose of prednisone was increased to 60 mg qd. In July 2020, a segment cesarean section was performed, whose procedure was successful, resulting a live infant. Following discharge, the patient was prescribed tacrolimus of 1 mg bid and prednisone of 70 mg qd. In January 2021, the patient received rituximab treatments on three occasions with doses of 0.1 g d1 and 0.5 g d2 initially, followed by 0.1 g d1 in subsequent two treatments. The prednisone dose was reduced to 40 mg qd after the first treatment and further decreased, respectively, to 30 mg qd and 25 mg qd in September 2021 and January 2022. MMF (1 g bid) was added in September 2022 but discontinued after 18 days. CsA (25 mg bid) was added in November 2022 but, later, discontinued after 14 days. AZA (50 mg qd) was added in March 2023 but discontinued in April 2023 when the fourth rituximab treatment was administered (0.1 g d1). In June 2023, the patient was prescribed telitacicept (160 mg quaque semana (qw)) and added efgartigimod (800 mg qw) in January 2024.

A 37-year-old female patient was diagnosed with MG in 2011 when she was first admitted to our hospital with clinical presentation of fluctuating ptosis, diplopia, upper and lower limb weakness, dyspnea, dysphagia, and dysarthria. Serology was positive for AChR-Ab (>20 nmol/L, reference value < 0.45 nmol/L) and RyR-Ab by enzyme-linked immunosorbent assay. Low-frequency repetitive nerve stimulation showed an abnormal amplitude decrease, and neostigmine test showed a positive result. Autoimmune-related antibodies, including antinuclear antibodies, anti-Sjögren's-syndrome-related antigen A (SSA) antibodies, and anti-SSB antibodies, all tested negative. By the way, she has no thymic pathology. Her clinical manifestation was well controlled by pyridostigmine (180 mg/day) in the early years. Since 2018, she had frequent exacerbations or myasthenic crises and required hospitalization for a pulmonary infection. In February 2022, she was hospitalized again with dyspnea due to pregnancy. Fortunately, she delivered a healthy baby. Throughout the multiple crises, IVIg, PLEX, and high-dose steroid treatment were indispensable, with endotracheal intubation to assist ventilation and a gastric tube, even a gastrostomy, to assist food and drug intake. Multiple immunosuppressants such as tacrolimus, rituximab (RTX), azathioprine (AZA), mycophenolate mofetil (MMF), and cyclosporine A (CsA) did not significantly improve symptoms. Consistent high doses of prednisone and pyridostigmine result in various complications such as hyperprolactinemia, hyperglycemia, hyperlipidemia, myelosuppression, lower limb venous thrombosis, and anal fistula. Since late June 2023, when she started receiving subcutaneous injections of telitacicept (160 mg/week), her symptoms of dyspnea and dysphagia have improved well, and her limb weakness is better than before. Her QMG (quantitative myasthenia gravis) and ADL (activity of daily living scale) scores have also decreased significantly to date (February 2024). Meanwhile, to further reduce the amount of prednisone, we combined it with efgartigimod (10 mg/kg, 800 mg/week) in January 2024. The details of the treatment regimens and drug doses are shown in **Figure 1**.

## 3 Treatment outcome and follow-up

**Figure 2:**
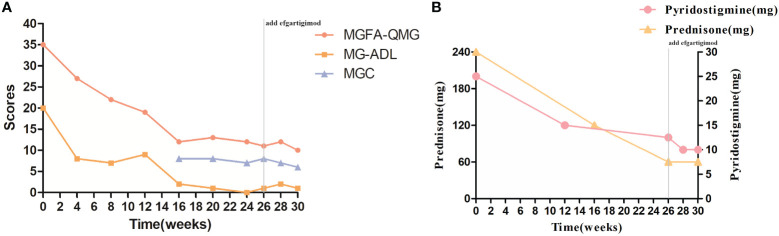
Evolution of clinical severity after injecting telitacicept. **(A)** Changes in MGFA-QMG, MG-ADL, and MGC. **(B)** Changes in levels of dose of prednisone and pyridostigmine. Abbreviations: QMG, quantitative myasthenia gravis score; MG-ADL, myasthenia gravis-specific activities of daily living scale; MGC, myasthenia gravis composite; W0, the week before the first injection of telitacicept (June 2024).

**Figure 3:**
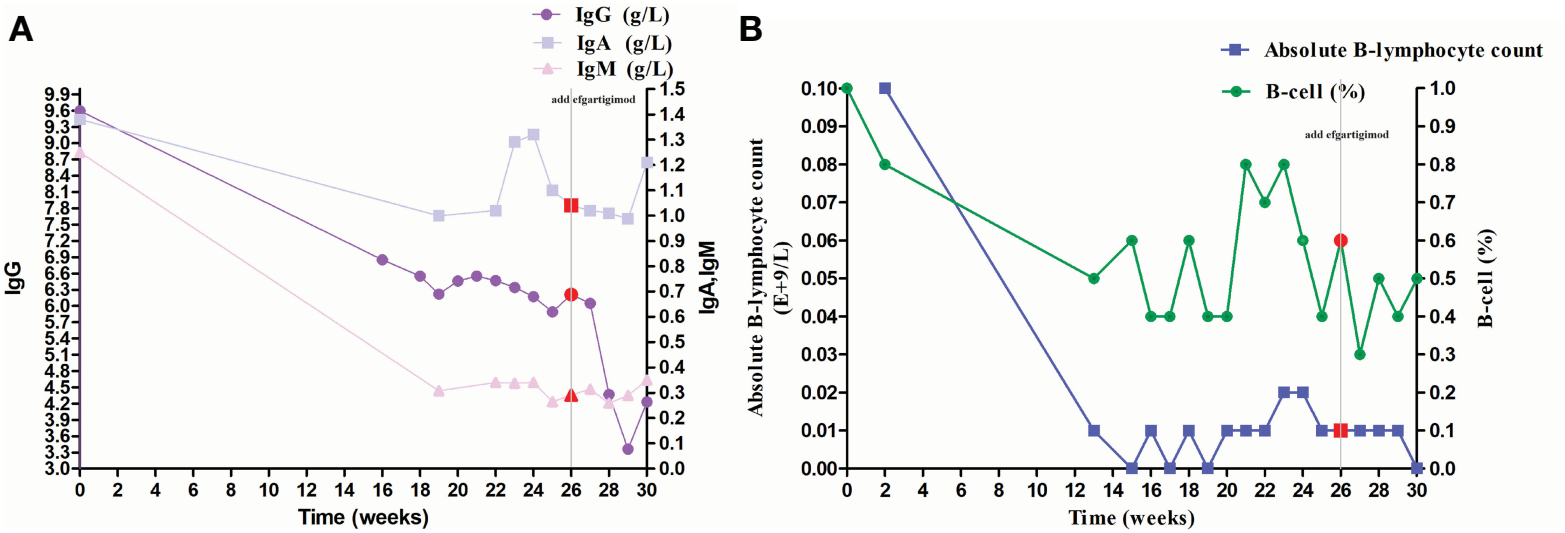
Changes in serum marker during follow-up. **(A)** Changes in immunoglobulin. **(B)** Changes in lymphocyte. The red point means the time of combination. IgG, immunoglobulin G.

The QMG and ADL scores have decreased significantly because the patient was injected with telitacicept for 4 weeks (decrease in QMG and ADL scores by 8 and 12 points, respectively), especially with regard to upper and lower limb weakness, dyspnea, and dysphagia. After 8 weeks of injections, prednisone was reduced to 15 mg/day (dose reduction of 10 mg/day), and pyridostigmine was reduced to 120 mg/day (dose reduction of 120 mg/day). The patient now attends the hospital weekly for regular telitacicept doses. In order to further reduce the dose of prednisone and pyridostigmine for less side effects, we combined it with efgartigimod in January 2024. By February 2024, she had received 31 regular injections of telitacicept (160 mg qw) and four regular injections of efgartigimod (800 mg qw), with a remarkable clinical improvement (QMG and ADL decreased to 10 and 1 points from baseline, respectively), and the doses of prednisone and pyridostigmine were reduced to 10 mg/day and 60 mg/day, respectively (**Figure 2**). During treatment with telitacicept and/or efgartigimod, symptoms were well controlled, and no exacerbation/myasthenic crisis occurred. In addition, serum B-cell, lymphocyte, and immunoglobulin levels showed a decreasing trend (**Figure 3**). With the combination of efgartigimod, the presentation of ptosis was well improved. However, the patient complained of hyperphagia after injection of efgartigimod and a slight swelling at the subcutaneous injection site, which disappeared in 1–2 days. In addition, hepatorenal function was within normal limits, and there were no infections or other adverse reactions.

## 4 Patient perspective

As a young woman with recurrent dyspnea and dysphagia who was diagnosed with MG 13 years ago, the previous multiple ineffective immunosuppressive therapies have greatly increased her anxiety, with invasive therapies such as PLEX and endotracheal intubation. Then, the stress of pregnancy and the complications of prolonged high-dose corticosteroids came. Fortunately, with the introduction of telitacicept and efgartigimod, her clinical presentation improved dramatically in terms of breathing and swallowing, and the dosage of steroids was gradually reduced. In the meantime, we have obtained individual informed consent and satisfaction with this combination therapy.

## 5 Discussion

### 5.1 Selection and deactivation of tacrolimus

Although the guideline and related research support that glucocorticosteroids and AZA are the basic immunotherapeutic agents for the treatment of mild/moderate to highly active GMG (4), AZA has a relatively slow therapeutic effect and side effects such as myelosuppression and liver injury. Therefore, we choose tacrolimus, which is faster than AZA, to combine with prednisolone. Compared with cyclosporine, tacrolimus is given in lower doses and has a lower nephrotoxic potential, although both are calcineurin inhibitors. It is suitable for patients who are unable to tolerate the side effects of prednisolone or other immunosuppressants to choose tacrolimus, especially patients with MG positive for RyR-Ab. It has been reported that intervention with calcineurin inhibitors is beneficial for early achievement of minimal manifestations (9). When she was diagnosed at 7 weeks of gestation in February 2020, with an urgent request to continue the pregnancy, we had to discontinue this inhibitor given the teratogenic potential of tacrolimus. Given the lack of clear guidelines or expert consensus on the treatment of MG during pregnancy, traditional therapies such as IVIg and PLEX are still being chosen. Fortunately, the patient had a smooth pregnancy and delivered a healthy baby by cesarean section. It has been found that the mothers who received immunotherapy during pregnancy, especially early and regular IVIG and/or PLEX, would have less severe complications and higher survival rate of offspring (10). Our case seems to confirm this point as well.

### 5.2 Choose of rituximab

RTX, a chimeric monoclonal antibody directed against the B-cell membrane marker CD20, acts through the mechanisms of complement- and antibody-dependent cytotoxicity (11, 12). Our therapy regimen with RTX was in regulation every 6 months (0.1 g D1 and 0.5 g D2) from January 2021, during which the B cells were kept at a low level. In addition, the dose of prednisone was reduced from 40 mg/day to 25 mg/day. However, the efficacy was not really significant, and the clinical symptoms still worsened repeatedly and recurrently. It has been reviewed that, despite initial responsiveness to RTX, many patients experience relapse during the reconstitution of B cells. This relapse can occur due to the re-encounter of antigens by RTX-resistant B cells or the reactivation of autoimmune responses by newly generated B cells during the process of B-cell repopulation (13). In exploring the reasons for the poor efficacy of RTX in this case, we contribute the following opinions. Firstly, as a B-cell depleting agent that directly targets CD20, RTX shows inadequate clearance of plasma cells, especially those long-lived plasma cells that continue to secrete antibodies that are located in the bone marrow or at sites of inflammation (14). Secondly, germinal center/memory B cells counteract the depletion of RTX by persistently producing pathogenic short-lived plasma cells, which are also involved in the pathogenesis of neurological autoimmune diseases. In addition, a study showed that the effects of RTX treatment were significantly better in patients with MuSK-MG than in those with AChR-MG (15).

### 5.3 Temporary attempt with MMF, CsA, and AZA

MMF selectively inhibits the proliferation of T and B lymphocytes by inhibiting enzymes involved in purine biosynthesis (16). In this case, the patient developed severe gastric reflux symptoms within half a month of taking MMF, and the drug was immediately discontinued in view of the patient’s gastrostomy status and the fear that the refluxed material would block the airway, causing respiratory distress or aspiration pneumonia. Compared to tacrolimus, CsA has a higher nephrotoxic potential and several other side effects such as hypertension, myalgia, and influenza-like symptoms (17). Unfortunately, the woman developed severe drug-induced liver injury with renal insufficiency and had to stop taking cyclosporin after 10 days. AZA is an effective therapeutic drug for all subtypes of MG that interferes with DNA synthesis by inhibiting the synthesis of purine nucleotides in rapidly proliferating T and B cells (16). After taking AZA for 1 month, the patient developed severe myelosuppression and a lung infection, so the drug was stopped immediately.

### 5.4 A new attempt with telitacicept

The main role of B cells in the body’s immune response involves complement activation, antigen presentation, antibody secretion, and cytokine release. Activated B cells can differentiate into plasma cells, which can further differentiate into short-lived plasma cells and long-lived plasma cells, thereby secreting a large amount of antibodies in a short period, leading to the recurrence of autoimmune diseases.

The BLyS and APRIL are critical factors in maintaining the B-cell pool and humoral immunity, and both play different roles during B-cell maturation and differentiation. BLyS inhibits the development and maturation of immature B cells and regulates their differentiation into mature B cells. APRIL affects the secretion of autoantibodies by autoreactive plasma cells by inhibiting the differentiation of mature B cells into plasma cells. Blocking both BLyS and APRIL can directly contribute to the reduction of plasma cells (18). Therefore, the B-cell targeted drug by blocking the maturation of plasma cells is a new approach in autoimmune diseases. Mono inhibition of BLyS or APRIL has little effect on plasma cell depletion (19). For example, belimumab, which interferes with the binding of BAFF to BCR, thereby inhibiting B-cell differentiation and preventing antibody production, showed a poor outcome in its phase 2 clinical trial (20, 21).

Telitacicept is a novel recombinant fusion protein of the ligand-binding domain of the TACI receptor and the Fc component of human IgG. By competitive inhibition of calmodulin cyclin ligand interaction factor (TACI) to neutralize BAFF and APRIL activity, it achieves multistage inhibition of B-cell and plasma cell maturation and differentiation (8). Meanwhile, due to the presence of TACI receptors on the surface of T cells, telitacicept also inhibits T-cell activation (8). In its phase 2 clinical trial, we find that telitacicept has good clinical efficacy and favorable safety in patients with GMG. Telitacicept has been shown to be a way to improve B-cell depletion and improve the safety and efficacy of RTX (22). Telitacicept targets BLyS/APRIL to inhibit the maturation and differentiation of B cells and plasma cells in multiple stages, thereby further suppressing the secretion of autoantibodies such as IgG. Therefore, compared to FcRn antagonists (e.g., efgartigimod) that directly act on IgG levels in the serum, telitacicept takes longer to take effect. The reasons for choosing telitacicept as a new attempt in June 2023, which takes effect relatively slower than efgartigimod, are as follows: 1. The half-life of IgG antibodies is approximately 16–24 days, meaning the maintenance period after clearing serum IgG with efgartigimod is short. Telitacicept targets inhibition from the upstream of the immune mechanism, resulting in a relatively longer maintenance period compared to efgartigimod. 2. During the last critical admission period in April 2023, the patient underwent rapid therapy of PLEX in the ICU. Considering that PLEX also works by clearing antibodies, complement, cytokines, and other substances in the plasma, similar to the mechanism of action of efgartigimod, telitacicept was chosen after this exacerbation. 3. At that time, efgartigimod has not yet been included in medical insurance coverage. Considering the patient’s 13-year illness history and comprehensive economic considerations, we have chosen telitacicept as the first choice for a new attempt.

In our case report, the woman experienced significant improvement after approximately 16 weeks of telitacicept (QMG and ADL scores decreased to 12 and 2 points, respectively) (**Figure 2**). In addition, serum levels of B cells, lymphocytes, and immunoglobulins showed a decreasing trend, demonstrating the efficacy of telitacicept (**Figure 3**). Finally, no infections or other adverse events were observed during the entire follow-up period, demonstrating the tolerability and safety of telitacicept, which are as important as efficacy from the patient’s perspective. Following the recent administration of RTX in early April 2023, which can result in peripheral B-cell depletion lasting approximately 6 months, the efficacy of RTX may persist until early October (around week15). Consequently, the potential role of RTX cannot be entirely discounted in the initial phase of telitacicept treatment. Further rigorous randomized controlled clinical trials are necessary to confirm the efficacy and safety of telitacicept.

### 5.5 The combination with efgartigimod

Due to enhancements in the medical insurance system, efgartigimod was officially incorporated into medical coverage in January 2024. To further reduce the amount of prednisone and even reduce the amount of pyridostigmine, we considered combining it with efgartigimod. In the pivotal phase 3 ADAPT trial of efgartigimod, efgartigimod was shown to improve muscle function and strength in all muscle groups, leading to the observed efficacy in participants with GMG (5). Research has shown that efgartigimod may serve as a potentially effective option for myasthenic crisis (23). The IgG antibody has a half-life of approximately 16–24 days. FcRn plays a direct role in the recycling and upkeep of IgG levels in the serum, leading to a faster onset of action. **Figure 3** illustrates a noticeable decrease in IgG levels and lymphocyte count especially following the administration of efgartigimod (highlighted by the red data point). Following the second administration of efgartigimod at week 28, we observed a halving of IgG levels compared to week 26 (**Figure 3**), with this decline persisting after the third injection. While a slight upward trend was noted at week 30, the IgG levels remained lower than those at week 26. Our patient’s blurred vision improved significantly after the combination of efgartigimod (QMG and ADL scores decreased to 10 and 1 points, respectively). Compared to PLEX, FcRn inhibitors are less invasive, more specific (particularly the removal of IgG), and independent (no effect on other therapeutic drug levels) (24). During acute phases, utilizing efgartigimod for its direct impact on IgG to rapidly take effect, inducing symptom relief, and then transitioning to telitacicept for sustained B-cell modulation may be a new combined approach.

## 6 Conclusion

Considering the pathogenesis of MG and the mechanism of telitacicept, despite its slower onset of action, it is reasonable to anticipate a promising future for telitacicept in MG treatment. Moreover, our research suggests that the combination of telitacicept with the faster-acting efgartigimod could represent an effective and safe therapeutic approach for highly active refractory MG. However, the sequencing of these two targeted agents and the treatment cost require careful consideration. Efgartigimod has a rapid onset of action and can more specifically remove IgG compared to PLEX, without affecting the levels of other therapeutic drugs, but its maintenance period is short. Telitacicept has a slower onset of action but a longer duration of effect. Considering the differences in the mechanisms of action of the two drugs mentioned above, we recommend prioritizing efgartigimod during the acute exacerbation phase, followed by maintenance therapy with telitacicept. During non-acute phases, telitacicept may be preferred initially, with the option to add efgartigimod if the treatment response is inadequate. The advantages and disadvantages of sequentially using telitacicept followed by FcRn versus FcRn followed by telitacicept are currently unclear due to the insufficient clinical data. Further large-scale studies are required to validate these approaches.

## Data availability statement

The original contributions presented in the study are included in the article/supplementary material. Further inquiries can be directed to the corresponding authors.

## Ethics statement

The studies involving humans were approved by Ethics Committee of The First Affiliated Hospital of Guangzhou University of Chinese Medicine. The studies were conducted in accordance with the local legislation and institutional requirements. The participants provided their written informed consent to participate in this study. Written informed consent was obtained from the individual(s) for the publication of any potentially identifiable images or data included in this article.

## Author contributions

CZ: Writing – original draft, Writing – review & editing, Investigation. YL: Writing – review & editing, Data curation, Investigation. QK: Writing – review & editing, Data curation, Investigation. HL: Writing – review & editing, Data curation, Investigation. QJ: Supervision, Writing – review & editing. XY: Supervision, Writing – review & editing.
